# Developmental Changes in Dynamic Functional Connectivity From Childhood Into Adolescence

**DOI:** 10.3389/fnsys.2021.724805

**Published:** 2021-11-22

**Authors:** Mónica López-Vicente, Oktay Agcaoglu, Laura Pérez-Crespo, Fernando Estévez-López, José María Heredia-Genestar, Rosa H. Mulder, John C. Flournoy, Anna C. K. van Duijvenvoorde, Berna Güroğlu, Tonya White, Vince Calhoun, Henning Tiemeier, Ryan L. Muetzel

**Affiliations:** ^1^Department of Child and Adolescent Psychiatry and Psychology, Erasmus MC University Medical Center, Rotterdam, Netherlands; ^2^The Generation R Study Group, Erasmus MC University Medical Center, Rotterdam, Netherlands; ^3^Tri-Institutional Center for Translational Research in Neuroimaging and Data Science (TReNDS), Georgia State University, Georgia Institute of Technology, Emory University, Atlanta, GA, United States; ^4^Barcelona Institute for Global Health (ISGlobal), Barcelona, Spain; ^5^Department of Molecular Genetics, Erasmus MC University Medical Center, Rotterdam, Netherlands; ^6^Department of Psychology, Harvard University, Cambridge, MA, United States; ^7^Leiden Institute for Brain and Cognition, Leiden University, Leiden, Netherlands; ^8^Department of Developmental and Educational Psychology, Leiden University, Leiden, Netherlands; ^9^Department of Radiology and Nuclear Medicine, Erasmus MC University Medical Center, Rotterdam, Netherlands; ^10^Department of Social and Behavioral Sciences, Harvard T.H. Chan School of Public Health, Boston, MA, United States

**Keywords:** brain development, fMRI, longitudinal, resting state – fMRI, linear mixed effect model

## Abstract

The longitudinal study of typical neurodevelopment is key for understanding deviations due to specific factors, such as psychopathology. However, research utilizing repeated measurements remains scarce. Resting-state functional magnetic resonance imaging (MRI) studies have traditionally examined connectivity as ‘static’ during the measurement period. In contrast, dynamic approaches offer a more comprehensive representation of functional connectivity by allowing for different connectivity configurations (time varying connectivity) throughout the scanning session. Our objective was to characterize the longitudinal developmental changes in dynamic functional connectivity in a population-based pediatric sample. Resting-state MRI data were acquired at the ages of 10 (range 8-to-12, *n* = 3,327) and 14 (range 13-to-15, *n* = 2,404) years old using a single, study-dedicated 3 Tesla scanner. A fully-automated spatially constrained group-independent component analysis (ICA) was applied to decompose multi-subject resting-state data into functionally homogeneous regions. Dynamic functional network connectivity (FNC) between all ICA time courses were computed using a tapered sliding window approach. We used a *k*-means algorithm to cluster the resulting dynamic FNC windows from each scan session into five dynamic states. We examined age and sex associations using linear mixed-effects models. First, independent from the dynamic states, we found a general increase in the temporal variability of the connections between intrinsic connectivity networks with increasing age. Second, when examining the clusters of dynamic FNC windows, we observed that the time spent in less modularized states, with low intra- and inter-network connectivity, decreased with age. Third, the number of transitions between states also decreased with age. Finally, compared to boys, girls showed a more mature pattern of dynamic brain connectivity, indicated by more time spent in a highly modularized state, less time spent in specific states that are frequently observed at a younger age, and a lower number of transitions between states. This longitudinal population-based study demonstrates age-related maturation in dynamic intrinsic neural activity from childhood into adolescence and offers a meaningful baseline for comparison with deviations from typical development. Given that several behavioral and cognitive processes also show marked changes through childhood and adolescence, dynamic functional connectivity should also be explored as a potential neurobiological determinant of such changes.

## Introduction

Neurodevelopment from childhood into adolescence represents a pivotal period, marked by several cognitive, social, and behavioral milestones, and is also beset with the emergence of many forms of psychopathology (Nelson et al., 2005; Paus et al., 2008; Luciana, 2013). Typical neurodevelopment provides a baseline framework for understanding how deviations in brain development are associated with mental and neurological illness, and it has been characterized *in vivo* using structural and functional magnetic resonance imaging (MRI) for over two decades (Giedd et al., 1999; Rubia et al., 2000; Luna et al., 2001; Gogtay et al., 2004; Lebel et al., 2008). More recently, resting-state functional MRI (rs-fMRI) has been used to study brain development. This brain imaging modality is used to measure intrinsic functional brain connectivity, or the spontaneous, correlated activations among brain networks (Biswal et al., 1995; Cole et al., 2010). The connectivity patterns of these networks exhibit high reproducibility between individuals, representing a reliable indicator of brain development (Allen et al., 2011). Despite widespread application, the vast majority of neurodevelopmental studies using rs-fMRI have been cross-sectional in design, lacking crucial insights from repeated measures (Kraemer et al., 2000).

Traditional rs-fMRI analysis approaches focus on the average functional connectivity across the scanning session, effectively assuming the connectivity is ‘static’. Studies of static brain connectivity have observed intra- and inter-network connectivity associations with age, and a number of networks show abnormal connectivity patterns in the presence of psychiatric disorders (Di Martino et al., 2014; Muetzel et al., 2016; Bos et al., 2017). Certain resting-state networks, such as the precuneus and the lateral frontal, increase their connectivity during brain development while others, such as the right frontoparietal and sensory networks, decrease with age (Muetzel et al., 2016). While static brain connectivity studies provide information about topological organization of functional brain networks during development, changes in connectivity throughout the scanning session are not captured by using this approach (Delamillieure et al., 2010; Calhoun et al., 2014).

Dynamic brain connectivity is a novel functional MRI analysis technique that allows connectivity between brain areas to vary over time, relaxing the stationarity assumption (Allen et al., 2014; Calhoun et al., 2014). Though several novel methods exist to estimate dynamic connectivity, one popular framework identifies different connectivity configurations, or states, across the scanning session and offers summary metrics, such as the time spent in each of these states. Although the general structure and topology of functional connectivity states are stable across age, there are age-related changes in the frequency of certain states and the time spent in each of them (Hutchison and Morton, 2015; Marusak et al., 2017). In the Generation R Study (*n* = 774, 6–10 years old), Rashid et al. (2018) found that older children spent more time in a state that showed a modular organization of functional connectivity in distinguished networks (Rubinov and Sporns, 2010), named ‘globally modularized dynamic state’. In this state, the nodes comprising a network were positively interconnected among them and those of different networks were negatively correlated. Contrarily, younger children spent more time in a globally disconnected state (Rashid et al., 2018). In addition, girls spent more time in a default mode modularized state compared to boys, which could indicate an earlier maturation of functional connectivity (Rashid et al., 2018). In the PING Study (*n* = 421, 3–21 years old), Faghiri et al. (2018) showed that age was negatively correlated with the time spent in states with strong connectivity between cognitive control and default mode domains, while older participants stayed longer in states showing positive intra-network connectivity within the default mode domain. Although the number of transitions between different states has not been associated with age in cross-sectional studies, some of these studies observed that the overall connectivity between intrinsic networks becomes more variable (higher standard deviation, SD) across the scanning session from childhood to adulthood (Hutchison and Morton, 2015; Qin et al., 2015; Marusak et al., 2017). For instance, Marusak et al. (2017) reported positive age associations with the variability of functional connectivity between core neurocognitive networks, which may afford greater cognitive and behavioral flexibility.

Currently, the literature examining associations between age and dynamic brain connectivity indicators in children has been comprised exclusively of cross-sectional studies. While these studies have established the fundamental basis for our understanding of age- and sex-related differences in functional brain connectivity, cross-sectional neurodevelopmental research provides limited information and it does not take into account inter-individual variability (Kraemer et al., 2000). Therefore, longitudinal studies are needed to explore individual growth changes, which is of key importance to understand the deviations in neurodevelopment after various exposures (e.g., early life adversities) or in psychopathology (Kraemer et al., 2000; Crone and Elzinga, 2015). Therefore, our objective was to characterize the longitudinal developmental changes in dynamic functional connectivity from childhood into adolescence in a large, population-based sample, as a follow-up of the cross-sectional findings observed in the Generation R Study by Rashid et al. (2018) at a younger age. We also aimed to understand whether maturation of dynamic functional connectivity was distinct in boys and girls, as sex differences have been observed using rs-fMRI (Satterthwaite et al., 2015) as well as other MRI modalities (Lenroot and Giedd, 2006; Perrin et al., 2009; López-Vicente et al., 2020). Our main focus was on global summary metrics of dynamic connectivity, specifically those related to time spent in a given connectivity configuration and the number of transitions between different connectivity configurations. Since previous cross-sectional studies suggested that the overall connectivity between intrinsic networks becomes more variable during development, we additionally tested the longitudinal age associations with the temporal variability (SD) of functional connections across the scanning session. We hypothesized that, over time, children would show more variable connections and they would spend more time in modularized states. This is in line with research indicating that brain development is characterized by the increase of “integration” of functional networks (Fair et al., 2007) and also with the existing dynamic connectivity literature (Faghiri et al., 2018; Rashid et al., 2018). Given differential developmental patterns previously reported with various neuroimaging modalities, we hypothesized that girls would show slightly faster development than boys, showing more mature dynamic connectivity patterns.

## Materials and Methods

### Participants

**FIGURE 1:**
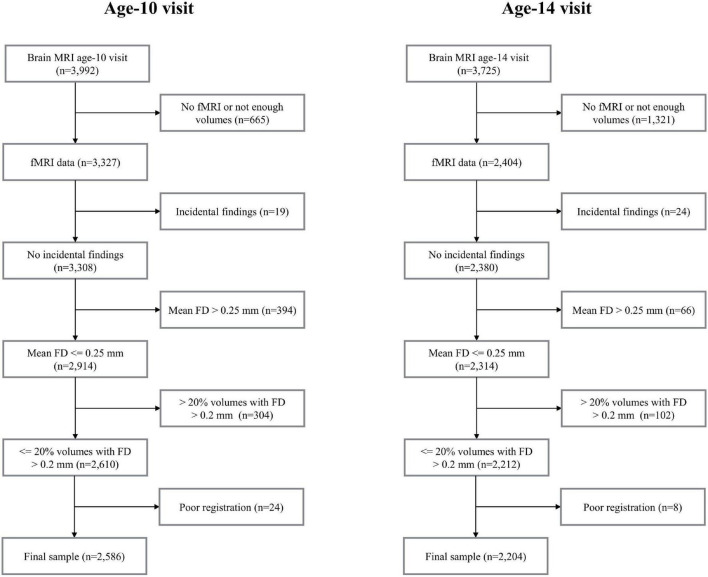
Flowcharts. MRI, magnetic resonance imaging; FD, framewise displacement.

The current study is part of the Generation R Study, a population-based birth cohort in Rotterdam, the Netherlands (Kooijman et al., 2016). Data in this study includes rs-fMRI data acquired at the age-10 visit (mean age 10 years, range 8-to-12, *n* = 3,327) and the age-14 visit (mean age 14 years, range 13-to-15, *n* = 2,404). Data collection was carried out between March 2013 and November 2015 for the age-10 visit (White et al., 2018) and between October 2016 and January 2020 for the age-14 visit. A flow-chart outlining data inclusion/exclusion for the study can be found in Figure 1. Few data were excluded due to the presence of prominent incidental findings (*n*_age10_ = 19 and *n*_age14_ = 24). Due to excessive motion, 698 datasets (21%) were excluded from the age-10 visit and 168 datasets (7%) were excluded from the age-14 visit (see Section “Image Quality Assurance” for details). Data were also excluded due to poor registration (spatial normalization) (0.7% from the age-10 visit and 0.3% from the age-14 visit). After this filtering, the total number of datasets available for analysis in this study was 2,586 at the age-10 visit and 2,204 at the age-14 visit. 1,031 participants had data in both visits (Figure 1). Supplementary Figure 1 shows the age at scan for all the individuals with repeated measures. All parents provided written informed consent and children provided assent (younger than 12 years) or consent (12 years or older). All study procedures were approved by the local medical ethics committee of the Erasmus MC University Medical Center.

### Magnetic Resonance Imaging Data Acquisition

Magnetic resonance images were acquired on a study-dedicated 3 Tesla GE Discovery MR750w MRI System (General Electric, Milwaukee, WI, United States) scanner using an 8-channel head coil. No hardware upgrades or major software upgrades have taken place since the study began in 2012 in order to keep the system stable for longitudinal research.

After a brief mock scanning session to acclimate the participants to the MRI environment, structural T_1_-weighted images were obtained using a 3D coronal inversion recovery fast spoiled gradient recalled (IR-FSPGR, BRAVO) sequence using ARC acceleration [*T*_R_ = 8.77 ms, *T*_E_ = 3.4 ms, *T*_I_ = 600 ms, flip angle = 10°, matrix = 220 × 220, field of view (FOV) = 220 mm × 220 mm, slice thickness = 1 mm]. 200 volumes of rs-fMRI data were acquired using an interleaved axial echo planar imaging sequence with the following parameters: *T*_R_ = 1,760 ms, *T*_E_ = 30 ms, flip angle = 85°, matrix = 64 × 64, FOV = 230 mm × 230 mm, slice thickness = 4 mm (White et al., 2018). The total duration of the resting-state scan was 5 min 52 s. Children were instructed to stay awake and keep their eyes closed.

### Image Preprocessing

Data were first converted from DICOM to Nifti format using dcm2niix (Li et al., 2016). Data were subsequently preprocessed through the FMRIPrep package (version 20.1.1 singularity container) (Esteban et al., 2019). Briefly, this included volume realignment for translation and rotation motion, slice-timing correction, and inter-subject registration.

Spatial normalization to the ICBM 152 Non-linear Asymmetrical template version 2009c (Fonov et al., 2009) was performed through non-linear registration with the antsRegistration tool of ANTs v2.1.0 (Avants et al., 2008), using brain-extracted versions of both T1w volume and template. The resulting functional data were resampled to 3 mm × 3 mm × 3 mm isotropic voxels.

From the volume realignment, we obtained the time series corresponding to the first temporal derivatives of the six base motion parameters (3 translations and 3 rotations), together with their quadratic terms, resulting in the total 24 head motion parameters (6 base motion parameters + 6 temporal derivatives + 12 quadratic terms) to be used as confound regressors (see below) (Satterthwaite et al., 2013).

### Group-Independent Component Analysis and Dynamic Functional Network Connectivity Analysis

Group-independent component analysis and dynamic functional network connectivity analyses were performed using the Group ICA Of fMRI Toolbox (GIFT) software^1^ (GroupICAT v4.0b) (Calhoun et al., 2001; Calhoun and Adalí, 2012) in MATLAB R2020a.

#### Group-Independent Component Analysis

Prior to analysis, the first 4 volumes of each subject were excluded to ensure magnetic stabilization. Resting-state data was decomposed into functionally homogeneous regions applying a spatially constrained group-independent component analysis (scICA) via the multi-objective optimization ICA with reference algorithm (Du and Fan, 2013). The scICA method is a fully automated approach which uses aggregate component maps from previous group ICA analysis as reference to estimate subject specific independent components. This technique has been previously used on adult studies (Salman et al., 2019; Du et al., 2020). Here we used 51 reference components derived from the Dev-CoG developmental imaging study, and grouped them into seven networks: subcortical (SC), auditory (AUD), sensorimotor (SM), visual (VIS), default-mode (DMN), cognitive control (CC), and cerebellar (CB) (Supplementary Figure 2 and Supplementary Table 1) (Agcaoglu et al., 2019).

The subject specific time courses corresponding to the components were detrended, despiked, and the 24 motion parameters were regressed out. As correlation among brain networks is primarily driven by low frequency fluctuations (Cordes et al., 2001), time courses were also filtered using a fifth-order Butterworth low-pass filter with a high frequency cut-off of 0.15 Hz.

#### Dynamic Functional Network Connectivity Analysis

Dynamic functional network connectivity (FNC) between all independent components time courses was computed using a tapered sliding window approach. This method provides multiple correlation matrices (one per window per dataset) that correspond to different temporal portions of data. We used a window size of 25 TR (44 s) in steps of 1 TR and the alpha parameter of the Gaussian sliding window was 3 TRs (Allen et al., 2014; Qin et al., 2015), which yielded 171 FNC windows per dataset. We estimated covariance from regularized inverse covariance matrices (Smith et al., 2011) using a graphical LASSO framework (Friedman et al., 2008) as estimation of covariance matrices of short time series can be noisy. Also, we imposed an additional L1 norm constraint on the inverse covariance matrix to enforce sparsity. The regularization parameter was optimized for each subject/visit by evaluating the log-likelihood of unseen data in a cross-validation framework, that is, splitting time courses into training and testing sets. Finally, to stabilize variance, the dynamic FNC values were Fisher-*Z* transformed.

#### Clustering

Using data from both visits, we computed k-means clustering on the resulting 171 dynamic FNC windows of 44 s from each scan session in order to identify patterns of connectivity that reoccur over time (within the scan session) and across subjects and visits (between the scan sessions). The number of clusters, or states, was set to five to match previous studies (Faghiri et al., 2018; Agcaoglu et al., 2020). We used the correlation distance function and the clustering algorithm was repeated 500 times to increase the chances of escaping local minima, with random initialization of centroid positions. We determined the modularity of the dynamic states qualitatively. First, a state was described as fully modularized when a clear modular organization, thus positive intra-network connectivity and negative inter-network connectivity, was observed. Next, if a state was not fully modularized, but presented sub-modules within networks with different connectivity configurations, we defined it as being partially modularized. Lastly, if a state did not possess any or very little characteristics of being modularized, we labeled it as a non-modularized state.

#### Outcome Measures

For each individual and visit, we calculated three different outcomes. First, the SD of the functional connections between the 51 components as a measure of temporal variability. Second, the mean dwell time (MDT) in each dynamic state. This variable was obtained by first identifying every change between states, calculating the number of windows in each state and computing the average time a participant remained in the specific states [for a more detailed explanation, see Rashid et al. (2018)]. Third, the number of transitions between states.

### Image Quality Assurance

Scans with excessive motion defined as having a mean framewise displacement (FD) higher than 0.25 mm or having more than 20% of the volumes with a FD higher than 0.2 mm, were excluded (Parkes et al., 2018). Image co-registration was visually inspected for accuracy by merging all co-registered images into a single 4D Nifti image and scrolling through the images. Scans were also screened for major artifacts (e.g., from dental retainers, or other scanner-related artifacts) as well as whole-brain coverage (e.g., missing from field of view). All the scans flagged as being of poor quality for the above-mentioned reasons were excluded.

### Sample Characteristics

Descriptive characteristics of the participants are presented with means and standard deviations or proportions. Child sex and date of birth were determined from medical records obtained at birth. Child ethnicity was defined based on the country of birth of the parents and was coded into three categories (Dutch, non-western, and other western). Maternal education level and household income, proxies of socioeconomic status, were assessed by questionnaire during pregnancy.

### Statistical Analyses

Statistical analyses were conducted using the R Statistical Software (version 3.6.0).

We compared the summary metrics (MDT in each state and number of transitions between states) between age-10 and age-14 visits and between boys and girls in each visit using Wilcoxon tests. For the visit comparison we used Wilcoxon signed rank test, while for the sex comparisons, we used Wilcoxon rank sum test.

Age was centered to the mean age of the sample at the age-10 visit. The distributions of all the dependent variables were visually inspected using histograms. Since MDT outcomes were right-skewed, we applied a Box–Cox transformation (Box and Cox, 1964) using the ‘bestNormalize’ package (version 1.6.1) (Peterson and Cavanaugh, 2020) to obtain a more homogeneous range of values.

The age associations with the temporal variability of connectivity (SD) between components, and the summary metrics (transformed MDT in each state and number of transitions between states) were estimated using linear mixed-effects models, implemented in the ‘nlme’ package (version 3.1-139) (Pinheiro et al., 2019), including age and sex as fixed effects and subject as random effect, allowing the intercept to vary randomly across subjects. For the summary metrics, we also tested the quadratic term of age in order to capture the possible non-linearity in the growth changes, and we added an interaction term of age-by-sex into the regression models to detect potential differential age associations in boys and girls, following a step-wise approach. We performed the likelihood ratio (LR) test for model comparisons using ML estimation. Stratified analyses by sex were performed when we observed statistically significant interactions. Given the important role of socioeconomic status in the brain development (Brito and Noble, 2014), we additionally included maternal education in the models as a precision covariate. The models were performed separately for each outcome. A false discovery rate (FDR) was applied to control for Type-I error. Associations with *p*_corrected_ < 0.05 were considered significant.

The associations between age and the summary metrics were graphically represented in the original scale using the ‘ggplot2’ package (version 3.3.2) (Wickham, 2016). To estimate the variation of the values in the population, we applied the bootstrap technique (Efron, 1979), using 2,000 resamples with replacement.

## Results

### Sample Characteristics

The sample characteristics are shown separately for participants with a single measurement (age-10 or age-14 visit) and those with repeated measurements in Table 1. The mean age and variation at the age-10 visit were very similar between those with and without repeated measurements, as well as at the age-14 visit. The mean duration between visits was 4 years (range 1–6 years). Although the proportion of boys and girls was balanced in the participants with data only at the age-10 visit, there were more girls than boys with single measurement at age-14 visit (53% girls vs. 47% boys) and with data at both visits (55% girls vs. 45% boys). Around 60% of the participants were of Dutch origin and between 26 and 31% were of non-western national origin, with small differences between single/repeated measurement groups. The groups also differed slightly in terms of maternal education and household income, although the relative proportions were constant between them. The participants with data only at the age-14 visit had the highest proportion of low (3%) or secondary (33%) maternal education and lower household income (<2,400€ per month, 23%).

**TABLE 1:** Sample characteristics.

	Single measurement		Repeated measurements
	Age-10 visit	Age-14 visit	Age-10 and -14 visits
N	1,555	1,173	1,031
Age (years, mean ± SD)	10.13 ± 0.58	14.18 ± 0.67	10.15 ± 0.62; 13.85 ± 0.51
*Sex (*n*, %)*			
Boys	787 (50.61)	546 (46.55)	466 (45.2)
Girls	768 (49.39)	627 (53.45)	565 (54.8)
*Ethnicity (*n*, %)*			
Dutch	948 (60.96)	671 (57.2)	642 (62.27)
Other western	138 (8.87)	107 (9.12)	94 (9.12)
Non-western	440 (28.3)	363 (30.95)	274 (26.58)
*Maternal education (*n*, %)*			
None or primary	38 (2.44)	30 (2.56)	24 (2.33)
Secondary	451 (29)	387 (32.99)	317 (30.75)
Higher	835 (53.7)	565 (48.17)	589 (57.13)
*Household income (*n*, %)*			
Very low (<1,200€/month)	83 (5.34)	52 (4.43)	40 (3.88)
Low (1,200€–2,400€/month)	235 (15.11)	215 (18.33)	168 (16.29)
Modal and higher (>2,400€/month)	950 (61.09)	666 (56.78)	675 (65.47)

*SD, standard deviation. The percentages of missing values in subjects with single measurement at age-10 visit were 2% for ethnicity, 15% for maternal education and 18% for household income. The percentages of missing values in subjects with single measurement at age-14 visit were 3% for ethnicity, 16% for maternal education and 20% for household income. The percentages of missing values in subjects with repeated measurements were 2% for ethnicity, 10% for maternal education and 14% for household income.*

### Temporal Variability in Functional Connections Within Scan Session

**FIGURE 2:**
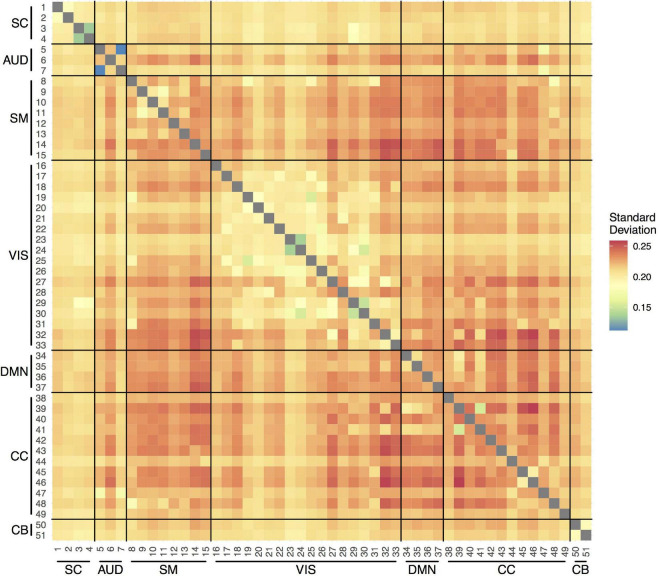
Temporal variability in pairwise functional connections between the 51 components within scan session. SC, subcortical network; AUD, auditory network; SM, sensorimotor network; VIS, visual network; DMN, default-mode network; CC, cognitive control network; CB, cerebellar network.

Figure 2 shows the average variability (SD) in the correlations between the time courses of the 51 components over the measurement period across participants and visits. In general, the SDs were between 0.20 and 0.25. The smallest variability was observed within the VIS network, indicating more stable connections over the scan session. The most variable connections were observed between SM and VIS networks, CC and VIS networks, and within the CC network.

### Dynamic States

**FIGURE 3:**
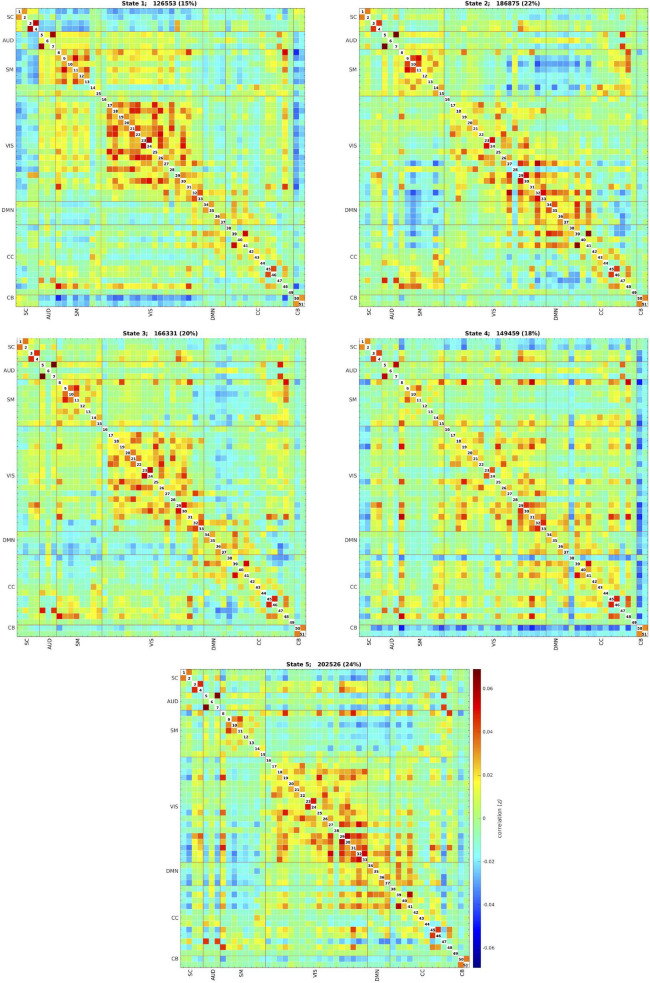
Five dynamic states after clustering across all individuals and visits. Each state captures a particular connectivity ‘configuration’ a participant may display over the course of the MRI scan. The total number and percentage of occurrences (windows of time) is listed above each state. SC, subcortical network; AUD, auditory network; SM, sensorimotor network; VIS, visual network; DMN, default-mode network; CC, cognitive control network; CB, cerebellar network.

**FIGURE 4:**
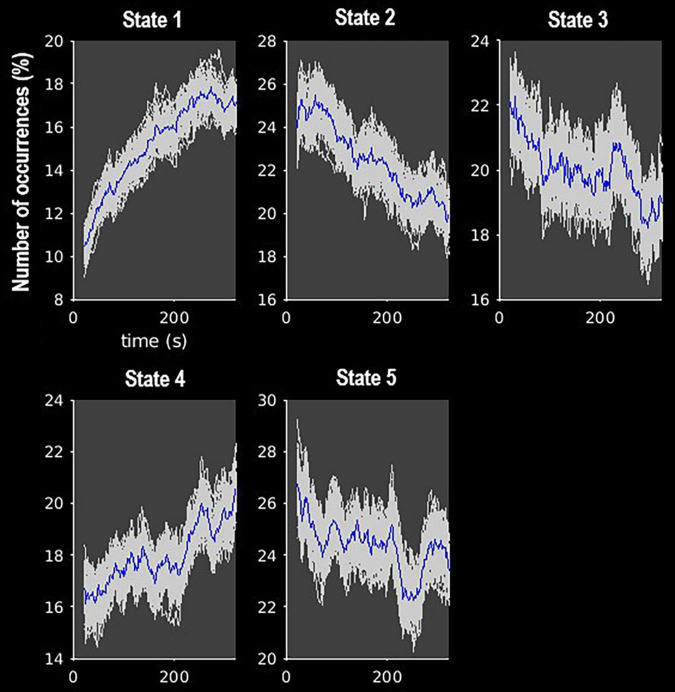
Frequency of occurrence of each state over the course of the MRI scan. Gray lines indicate the estimated occurrence profiles of each state for 100 bootstrap resamples with replacement.

The *k*-means clustering method allowed us to identify five dynamic states, or patterns of connectivity that reoccurred over time (within the scan session) and across subjects and visits (between the scan sessions). We obtained three modularized states with components showing intra- and inter-network connectivity (states 1, 2, and 3) and two non- or only partially modularized states (states 4 and 5) (Figure 3). In state-1 (15% of occurrences), the SC and CB networks showed positive intra-network connectivity and negative inter-network connectivity, mainly with the sensory networks (AUD, SM, and VIS). Thus, the components that comprise the SC and CB networks were positively correlated within themselves, and they were negatively correlated with the components of the sensory networks. The SM and the VIS networks also showed strong positive intra-network connectivity. The frequency of occurrence of state-1 increased with scan progression (i.e., occurred more as the scan went on, particularly, around 7% more windows of time were part of this state at the end of the session as compared to the beginning) (Figure 4). In state-2 (22% of occurrences), SM and DMN networks showed positive intra-network connectivity and negative inter-network connectivity between them. Other components, such as CC components, showed opposite connectivity patterns with SM and DMN. For instance, frontal CC components were positively correlated with DMN and negatively correlated with SM components, and posterior CC components showed the opposite pattern. The frequency of state-2 decreased by 5% with scan progression (Figure 4). State-3 (20% of occurrences) was characterized by positive correlations within the DMN and negative correlations between this network and the other networks, except some CC components from the frontal lobe. The SM and the VIS networks also showed strong positive intra-network connectivity in this state. State-3 shared some traits with state-2. However, the negative inter-network connectivity between DMN and the other networks was more “global,” although weaker, in state-3 than in state-2, in which the inter-network connectivity was focused on specific networks. The frequency of state-3 also decreased by 3% of windows of time over the scan session (Figure 4).

State-4 (18% of occurrences) was non-modularized, thus a clear modular organization of functional connectivity in distinguished networks was absent in this state. The putamen (SC, 1 and 2), the middle temporal gyrus (CC, 38), and the cerebellum were negatively correlated with all the other components. The opposite was observed in the postcentral gyrus component (SM, 8), which was positively correlated to several components. The frequency of state-4 increased by 4% with scan progression (Figure 4).

Finally, state-5 (24% of occurrences) was partially modularized, presenting sub-modules within networks with different connectivity configurations. For instance, regarding the SC network, the putamen (SC, 1 and 2) was negatively correlated with visual components, while the thalamus (SC, 3 and 4) showed positive correlations. The postcentral gyrus component (SM, 8) was positively correlated with visual components, and the rest of the SM components were negatively correlated with those components. As in state-3, the DMN was positively connected within network and with frontal CC components. The frequency of state-5 showed a general mild decreasing trend over the scan session (Figure 4).

### Longitudinal Changes in the Temporal Variability

**FIGURE 5:**
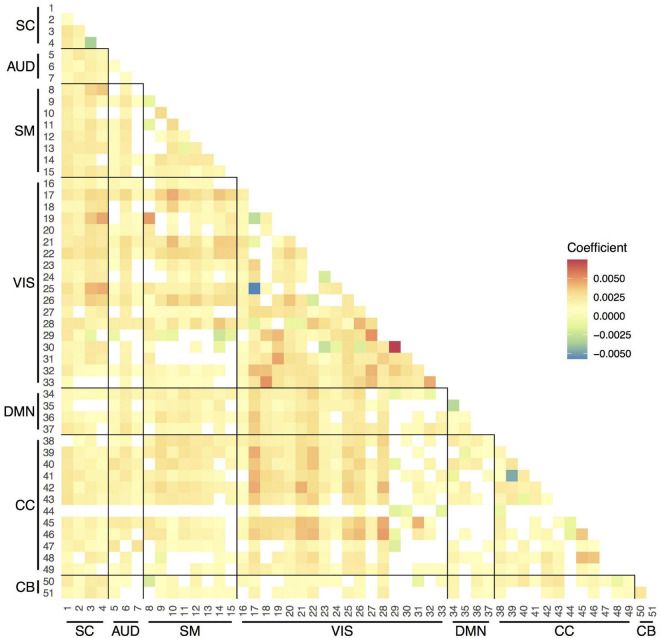
Strength of the associations between age and variability in pairwise functional connections between components. Linear mixed-effects models adjusted for sex (random effect: subject). Only the results that survived the false discovery rate (FDR) multiple comparison correction threshold of pFDR = 0.05 are shown here. SC, subcortical network; AUD, auditory network; SM, sensorimotor network; VIS, visual network; DMN, default-mode network; CC, cognitive control network; CB, cerebellar network.

We observed increases in overall temporal variability in functional connections between components across age (Figure 5). The SD, which ranged between 0.20 and 0.25, increased on average a 1% (coefficient = 0.0025) per year. Some connections showed less variability at older ages, such as the Insular Cortex component (CC, 44) connections with components from other networks.

### Longitudinal Changes in Dynamic States

**FIGURE 6:**
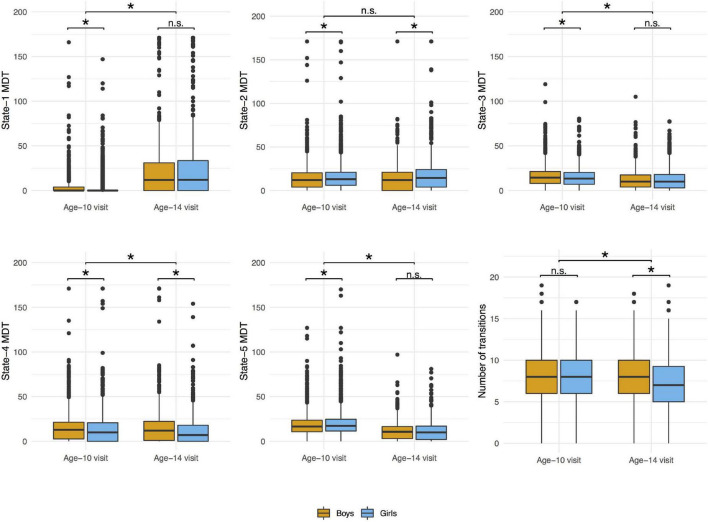
Distributions of mean dwell time (MDT, number of time windows) in each state and number of transitions between states by visit and sex. Wilcoxon signed rank test was used to compare the values between age-10 and age-14 visits and Wilcoxon rank sum test was used to compare the values between boys and girls in each visit. *p-value < 0.05; n.s., non-significant.

The distributions of the summary metrics (MDT and number of transitions) by visit and sex are depicted in the original scale in Figure 6, and the individual observations are shown in Supplementary Figure 3. We observed differences between age-10 and age-14 visits in all the outcomes except state-2 MDT. Boys and girls also showed differences in the MDT of states 1, 3, and 5 (age-10 visit), states 2 and 4 (both visits), and the number of transitions between states (age-14 visit). As outlined in the Section “Statistical Analyses”, the linear mixed-effects models were performed using transformed MDT outcomes (Box–Cox transform). Table 2 shows the age and sex associations with the MDT of each state and the number of transitions between states. Only the linear term of age was included in the models because the LR test indicated that the model fit was not significantly improved with the quadratic term addition. Overall, the MDT in state-1 increased with age, while the MDT in states 3, 4, and 5 decreased with age. The number of transitions between states decreased over time. Girls spent more time in state-2, less time in states 3 and 4, and showed fewer transitions between states compared to boys.

**TABLE 2:** Age- and sex-associations with transformed mean dwell time (MDT, number of time windows) in each state and number of transitions (NT) between states.

	Age				Sex (ref. boys)				AIC	BIC
		95% CI				95% CI				
	Estimate	Lower	Upper	*P*-value	Estimate	Lower	Upper	*P*-value		
State-1 MDT	0.208	0.196	0.221	< 0.001*	–0.052	–0.104	–0.001	0.046	12678.65	12711.02
State-2 MDT	0.001	–0.013	0.015	0.872	0.152	0.096	0.209	< 0.001*	13594.54	13626.9
State-3 MDT	–0.082	–0.096	–0.068	< 0.001*	–0.074	–0.130	–0.018	0.009*	13480.18	13512.55
State-4 MDT	–0.036	–0.049	–0.022	< 0.001*	–0.244	–0.302	–0.186	< 0.001*	13476.86	13509.23
State-5 MDT	–0.171	–0.185	–0.158	< 0.001*	0.040	–0.014	0.093	0.145	13003.42	13035.78
NT	–0.178	–0.221	–0.134	< 0.001*	–0.292	–0.468	–0.115	0.001*	24485.35	24517.71

*Linear mixed-effects models (random effect: subject). The MDT outcomes were transformed using Box–Cox. Age was centered to the mean age of the sample at age-10 visit.*

*P-value corrected for multiple comparisons (FDR) < 0.05. AIC, Akaike Information Criterion; BIC, Bayesian Information Criterion.

**FIGURE 7:**
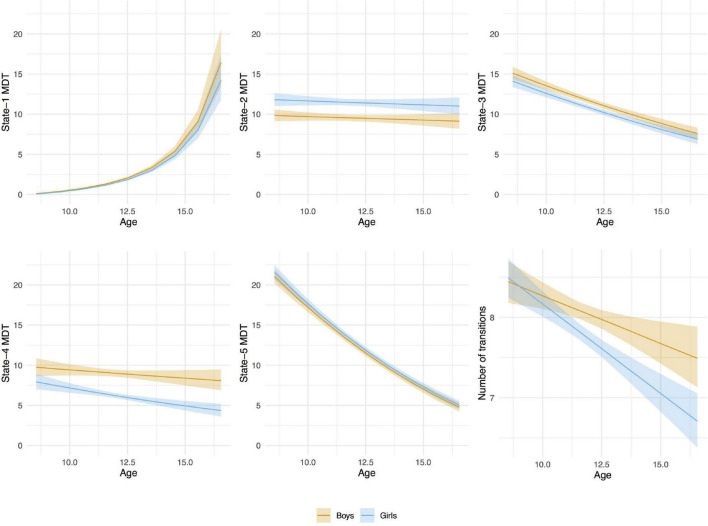
Age-associations with mean dwell time (MDT, number of time windows) in each state and number of transitions between states by sex. Linear mixed-effects models (random effect: subject). The MDT values were transformed back to the original scale for the graphical representation of the associations. A bootstrap technique was applied using 2,000 resamples with replacement to estimate the variation of the values in the population.

From baseline to follow-up, increases were observed for the time spent (MDT) in state-1, which is characterized by negative inter-network connectivity between subcortical and sensorimotor networks. Thus, children spent more time in state-1 as they grew older. Figure 7, depicts the predicted number of windows spent in state-1 increased slightly more in absolute terms at older ages than at younger ages. No sex differences were observed in MDT for state-1, and adding the age-by-sex interaction term did not improve the model (LR test *p* = 0.491). Regarding state-2 (the default-mode/sensorimotor modularized), we found differences by sex, with girls spending around 1% more time (2 windows of time) than boys in this state across the whole age range (Figure 7). However, no significant interaction was observed (LR test *p* = 0.051).

We observed decreases in MDT for state-3 (the default-mode network modularized state), state-4 (the non-modularized state), and state-5 (the partially modularized state) associated with age. Children spent around 0.6% less time (1 window) per year in state-3. In addition, girls spent around 0.6% less time (1 window) in this state than boys across the whole age period, with no age-by-sex interaction (LR test *p* = 0.120) (Figure 7). Regarding state-4, we found an interaction between age and sex (LR test *p* = 0.006) (Table 3 and Figure 7). The stratified analyses showed that the negative association between age and MDT in state-4 was stronger in girls (Table 4). Girls spent 0.3% less time (half window) per year in this state. In boys, the slope decreased more slowly with age (Figure 7). The change in state-5 was steeper, MDT decreased by 1% (2 windows) per year both in boys and in girls, with no age-by-sex interaction (LR test *p* = 0.361) (Figure 7).

**TABLE 3:** Age-, sex-, and age-by-sex associations with transformed mean dwell time (MDT, number of time windows) in each state and number of transitions (NT) between states.

	Age				Sex (ref. boys)				Age*sex interaction					
		95% CI				95% CI				95% CI			AIC	BIC
	Estimate	Lower	Upper	*P*-value	Estimate	Lower	Upper	*P*-value	Estimate	Lower	Upper	*P*-value		
**State-1 MDT**	0.203	0.185	0.222	< 0.001*	–0.068	–0.136	0.000	0.050	0.009	–0.016	0.034	0.492	12687.03	12725.87
**State-2 MDT**	–0.014	–0.034	0.007	0.187	0.103	0.028	0.178	0.007*	0.028	0.000	0.056	0.051	13599.4	13638.24
**State-3 MDT**	–0.094	–0.114	–0.073	< 0.001*	–0.113	–0.187	–0.039	0.003*	0.022	–0.006	0.049	0.121	13486.46	13525.3
**State-4 MDT**	–0.016	–0.035	0.004	0.116	–0.179	–0.254	–0.104	< 0.001*	–0.037	–0.063	–0.010	0.006*	13478.19	13517.03
**State-5 MDT**	–0.165	–0.184	–0.146	< 0.001*	0.061	–0.009	0.132	0.090	–0.012	–0.039	0.014	0.361	13011.37	13050.21
**NT**	–0.120	–0.184	–0.056	< 0.001*	–0.102	–0.337	0.132	0.391	–0.107	–0.194	–0.020	0.016*	24485.94	24524.78

*Linear mixed-effects models (random effect: subject). The MDT outcomes were transformed using Box–Cox. Age was centered to the mean age of the sample at age-10 visit.*

*P-value corrected for multiple comparisons (FDR) < 0.05. AIC, Akaike Information Criterion; BIC, Bayesian Information Criterion.

**TABLE 4:** Sex stratified age-associations with transformed mean dwell time (MDT, number of time windows) in state-4 and number of transitions (NT) between states.

		95% CI		
	Estimate	Lower	Upper	*P*-value
*State-4 MDT*				
Boys	–0.016	–0.035	0.004	0.111
Girls	–0.053	–0.071	–0.034	<0.001
*NT*				
Boys	–0.120	–0.184	–0.056	<0.001
Girls	–0.227	–0.286	–0.168	<0.001

*Linear mixed-effects models (random effect: subject). The MDT outcome was transformed using Box–Cox. Age was centered to the mean age of the sample at age-10 visit.*

The number of transitions between states decreased over time, and this association was stronger in girls than in boys, with age-by-sex interaction (LR test *p* = 0.016) (Tables 3, 4). The predicted number of transitions changed from NT = 8.5 around age-9 to NT = 7 at age-14 in girls (Figure 7).

Similar results were observed in the models that were additionally adjusted for maternal education (Supplementary Table 2).

## Discussion

This is the largest longitudinal population-based study describing individual changes in dynamic brain connectivity from childhood into adolescence. We highlight three findings that show developmental patterns. First, we found a general increase in the variability of the connections between intrinsic connectivity networks with increasing age. Second, the time spent in a modularized state increased with age, while the time spent in less modularized states decreased with age. Third, the number of transitions between states decreased with age. Girls showed a more mature pattern of dynamic brain connectivity, spending more time in a highly modularized state, less time in specific states that were more frequently observed at a younger age, transitioning less between states and showing a faster decrease of time spent in a non-modularized state across age than boys.

The higher variability in the connections between networks observed with increasing age is consistent with previous cross-sectional studies (Hutchison and Morton, 2015; Marusak et al., 2017). This broader repertoire of functional connections between brain regions could be a neural substrate of a higher cognitive complexity. Some of our findings regarding the associations between age and the time spent in specific dynamic states are consistent with previous research. Using cross-sectional data of the Generation R Study, but a younger age visit than this study (6–10 years old), Rashid et al. (2018) also found that older children showed longer MDT in a globally modularized state, characterized by intra- and inter-network connectivity. We found negative age associations with MDT in state-3, in which the DMN was negatively correlated with the other networks. We expected this type of connectivity pattern to be positively associated with age, given the modularity of the state and the fact that the efficiency of the DMN increases as children grow older. It is possible that state-3 is a precursor of state-2, and older children tend to spend less time in state-3 because they transition to the other modularized states (1 and 2).

We reported negative associations between age and time spent in less modularized states, such as states 4 and 5. These results are supported by previous research showing that the developing brain is characterized by an increase of “integration” of functional networks (Fair et al., 2007). The components of a network in those states do not show consistent intra-network connectivity, nor inter-network connectivity. This suggests that the integration, or the increased connectivity within the brain regions that comprise a network, is low. This is expected given adolescence is a period of transition to more efficient brain connectivity, in which widely distributed areas are integrated into complex brain systems. This type of developmental process, in which connections change during adolescence, has recently been identified as “disruptive mode,” in contrast to “conservative mode,” in which connections already established become more strong (Váša et al., 2020). Myelination and synaptic pruning processes that take place during brain development likely contribute to these changes in functional connections by supporting more efficient neuronal communication. The establishment of these complex functional systems has an impact on higher-order cognition (Giedd et al., 1999; Luna and Sweeney, 2004; Bunge and Wright, 2007; Fair et al., 2007).

We found negative associations between age and the number of transitions between dynamic states. Previous studies did not find such association during rest (Hutchison and Morton, 2015; Marusak et al., 2017). Our findings were statistically significant, however, the change we observed was relatively small. Given the large size of the current sample, the discrepancy in findings could be explained by the higher power of our study. Overall, our findings suggest that older participants show more complex connectivity patterns and they remain longer in specific connectivity configurations.

In terms of the composition of the different dynamic states, or configurations, some of the connections between components were stable between states, such as intra-network connectivity within the SC and the VIS networks. Consistent with previous work, this finding suggests that network organization in humans is a combination of both static and dynamic connections (Calhoun et al., 2014; Faghiri et al., 2018). The age-related changes in the dynamic connectivity metrics reported in this study indicate that the organization of human connectivity patterns develop progressively across the age spectrum (Faghiri et al., 2018). The largest change in MDT associated with age was observed in state-1. This connectivity configuration resembles one that has previously been identified as a “drowsiness pattern” (Allen et al., 2014; Damaraju et al., 2014, 2020). Interestingly, the frequency of this state increased with the scan progression, which could indicate an increase in the fatigue or a decrease in the anxiety of the participants along the session. At the same time, other states that are likely related to a higher awareness, such as state-2, showed the opposite pattern, its frequency decreased with scan progression. The detection of this drowsiness-related state could be beneficial for other rs-fMRI studies, since it allows to remove the effect of potential drowsiness from the data. Additionally, it could also prove interesting from a clinical perspective, where a particular disorder shows differential associations within this connectivity configuration.

Overall, girls showed a faster development of dynamic connectivity than boys. This is consistent with previous literature (Satterthwaite et al., 2015; Rashid et al., 2018) and may be due to an earlier onset of puberty in girls. We observed that girls spent more time than boys in the default-mode/sensorimotor modularized state (state-2) state across the whole age range. In this state, the DMN and the SM showed opposite activation patterns and they were negatively correlated between them; when one of those networks is activated, the other is deactivated. The DMN has been linked to internally focused thought, episodic memory, and planning the future (Buckner et al., 2008), while the SM is related to the processing of external stimuli and motor information. Hence, girls were more prone than boys to show a connectivity configuration in which the DMN and SM networks were negatively correlated, which could indicate a higher efficiency in the synchronized activation and deactivation of those networks. Similar patterns of modular organization between sensory systems and DMN have been observed in young adults (Allen et al., 2014). Faghiri et al. (2018) using cross-sectional data from a broader age range (3–21 years old), observed that older participants spent more time in states showing intra-network connectivity within the DMN, and inter-network connectivity between the SM and CC networks. These connectivity traits were similar to the ones of the default-mode/sensorimotor modularized state (state-2) that we obtained. However, in our study we did not find associations between age and the time spent in this state. Girls showed 1 year of advantage in state-3 MDT decrease in relation to boys and they showed a faster MDT decrease across age in the non-modularized state-4, more commonly observed in younger children. In addition, girls transitioned less between states than boys, which also suggests more mature connectivity.

One of the most important limitations of developmental fMRI studies is motion (Power et al., 2012). In order to reduce the impact of motion, we implemented various strategies at several levels of the analysis. First, we excluded datasets with excessive motion applying a strict threshold (Parkes et al., 2018). Second, we used a spatially constrained group-independent component analysis approach, only including components that were identified as not being noise. Third, we added a standard set of motion regressors to the dynamic FNC analysis (Satterthwaite et al., 2013). Indeed, there was no relationship between age and motion in our sample after excluding the datasets with excessive motion. The drawback of these actions is that participants who move more are underrepresented in the analyses, potentially leading to selection bias. In fact, we observed some differences between the participants with data only at the age-10 visit, those with data only at age-14 visit, and those with repeated measurements. The proportion of girls was higher in the second visit, the socioeconomic status was lower in the participants with data only at the second visit, while a higher socioeconomic level was more common among those with data at the two time points. Despite these small differences in the socioeconomic status between the groups, the inclusion of maternal education in the models as a precision variable did not change the results. Future work should explore the role of socioeconomic status on the development of dynamic connectivity. For example, socioeconomic status has proven an important factor in structural neurodevelopment (Brito and Noble, 2014). In this study, we used a group ICA template generated from a model order of 150, however, analyzing dynamic FNC with higher and lower group ICA model-based templates would also be interesting. Indeed, a full multi-spatial scale FNC analysis appears to provide additional information (Iraji et al., 2021). In terms of the tapered sliding-window approach used in this study, one limitation is related to the selection of the window size. However, it has been demonstrated that 44 s provides reliable connectivity estimations and it is also sensitive to abrupt brain activity (Allen et al., 2014; Qin et al., 2015). In addition, the observations were weighted according the their position within the window to avoid the effect of influential points (Allen et al., 2014). Another relevant limitation of this study was the distribution of the MDT outcomes with a relatively skewed distributions. Despite the Box–Cox transformation, the residuals from the linear mixed model for state-1 were not fully normally distributed. However, different transformations as well as not transforming the data at all, yielded highly similar results, and the large sample size of this study ensures the robustness of the estimates obtained even in non-ideal conditions (Knief and Forstmeier, 2021).

The longitudinal design, the large and multiethnic sample, which was based on the general population, and the use of a single MRI scanner are the main strengths of this study. Longitudinal studies are key to study the development of the brain, since they allow to control for interindividual variability (Kraemer et al., 2000). The advantages of studying the brain at the population level as opposed to using small samples include the higher statistical power, the lower bias and the higher generalizability of the results (Paus, 2010; White et al., 2013; LeWinn et al., 2017). The use of a single scanner is important as it reduces vendor- and hardware-dependent differences, and it avoids the possible influence of the system updates on the longitudinal estimates.

To summarize, we observed longitudinal changes in dynamic connectivity from ages 8–15 years. Particularly, as children mature, they show: (1) a higher variability in the connections between networks; (2) less time in less modularized states; and (3) less transitions between states. Girls showed a more mature pattern of dynamic connectivity. Resting-state functional connectivity is a reliable tool for studying functional neurodevelopment as it does not require an explicit task-based framework and the connectivity of intrinsic networks exhibits high reproducibility between individuals. Dynamic brain connectivity approaches offer a more comprehensive view of functional connectivity than static connectivity alone and they provide summary metrics, which are likely more reproducible than many thousands of individual edge comparisons. In conclusion, the changes of dynamic connectivity over the course of development presented in this work provide a meaningful baseline for comparison in deviations from typical development.

## Data Availability Statement

The datasets presented in this article are not readily available because of legal and ethical regulations. Requests to access the datasets should be directed to Vincent Jaddoe, v.jaddoe@erasmusmc.nl.

## Ethics Statement

The studies involving human participants were reviewed and approved by the local medical ethics committee of the Erasmus MC University Medical Center, Rotterdam, Netherlands. Written informed consent to participate in this study was provided by the participants’ legal guardian/next of kin.

## Author Contributions

ML-V and RLM contributed to conception and design of the study and wrote the first draft of the manuscript. RLM preprocessed the data. ML-V, LP-C, FE-L, RHM, JF, and RLM contributed to data curation. ML-V, OA, JH-G, VC, and RLM contributed to methodology and software. ML-V performed the analyses. All authors contributed to manuscript revision, read, and approved the submitted version.

## Conflict of Interest

The authors declare that the research was conducted in the absence of any commercial or financial relationships that could be construed as a potential conflict of interest.

## Publisher’s Note

All claims expressed in this article are solely those of the authors and do not necessarily represent those of their affiliated organizations, or those of the publisher, the editors and the reviewers. Any product that may be evaluated in this article, or claim that may be made by its manufacturer, is not guaranteed or endorsed by the publisher.

## Footnotes

1
https://trendscenter.org/software/gift
