# Supplementary material for: Developmental Changes in Dynamic Functional Connectivity From Childhood Into Adolescence
Source: Front Syst Neurosci. 2021 Nov 22;15:724805. doi: 10.3389/fnsys.2021.724805 (PMC8645798; doi:10.3389/fnsys.2021.724805)
Supplement: Supplementary file 1 [file Data_Sheet_1.pdf]

Supplementary Material

Supplementary Figure 1. Age at scan for individuals with repeated measures. The participants are ordered based on their age at the first visit.

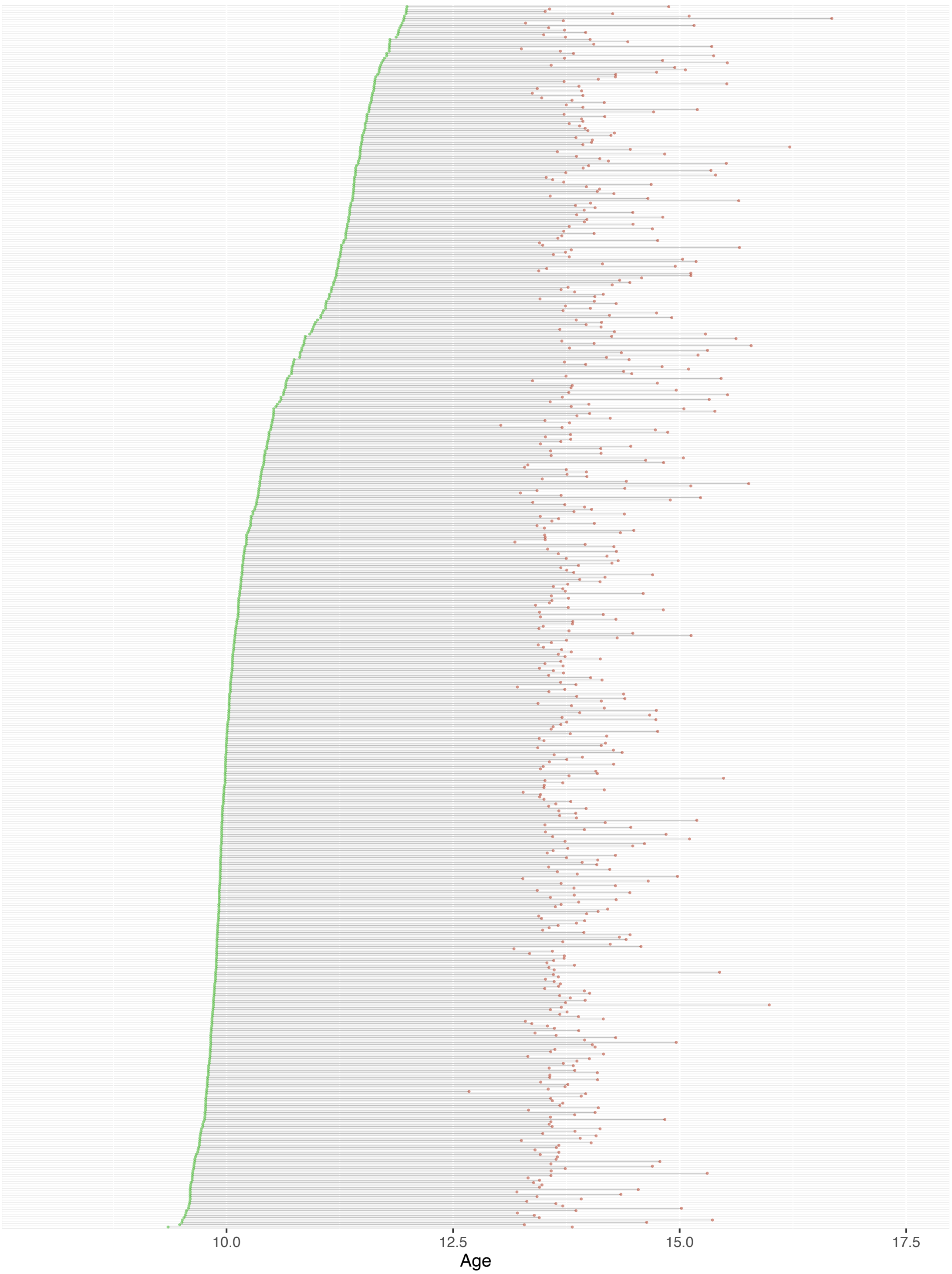

Supplementary Figure 1. Continued.

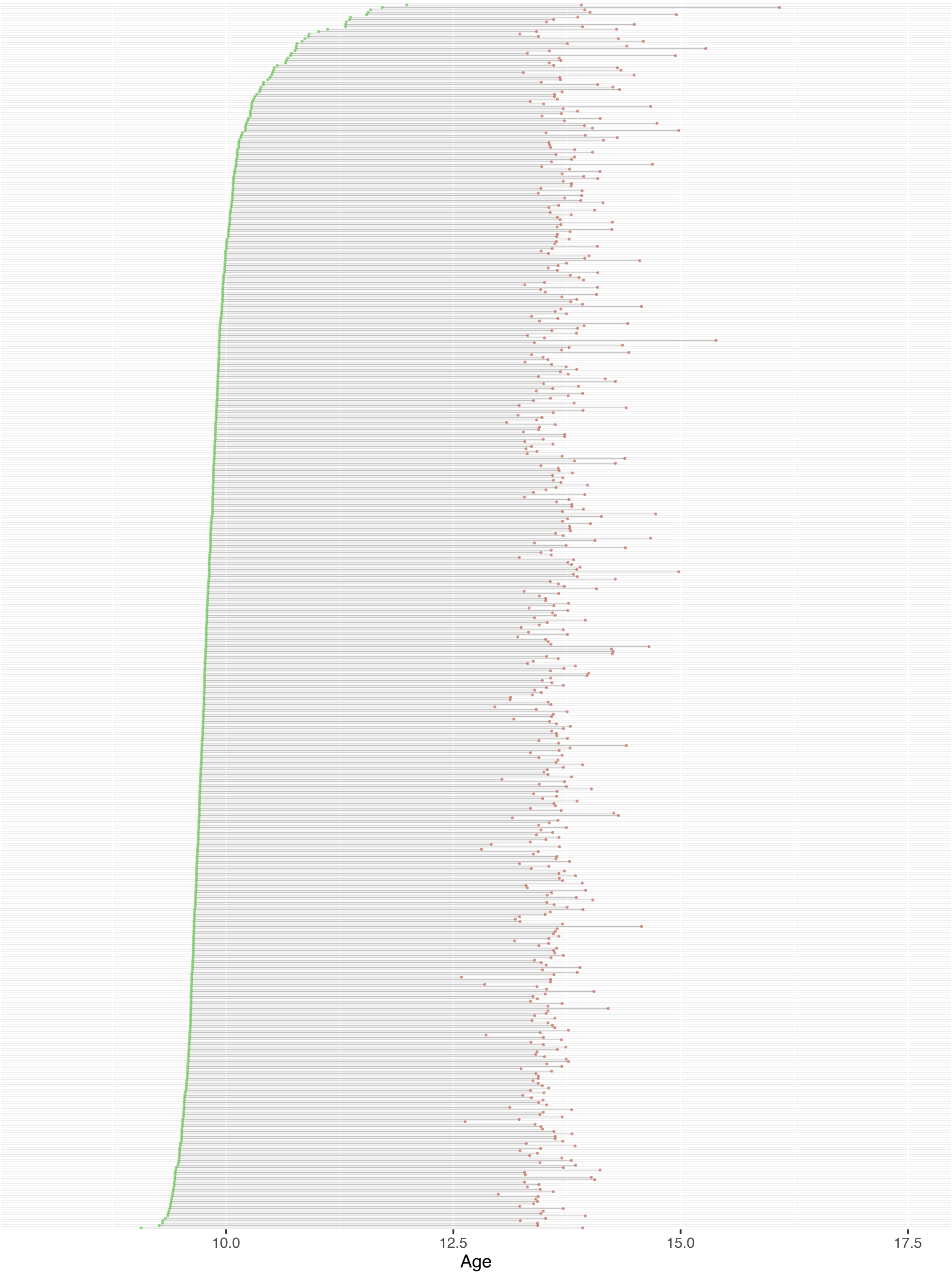

Supplementary Figure 2. The 51 resting state components grouped by networks, including 4 subcortical (SC), 3 auditory (AUD), 8 sensorimotor (SM), 18 visual (VIS), 4 default-mode (DMN), 12 cognitive control (CC), and 2 cerebellar (CB), estimated using the component maps from the Dev-CoG developmental imaging study as reference

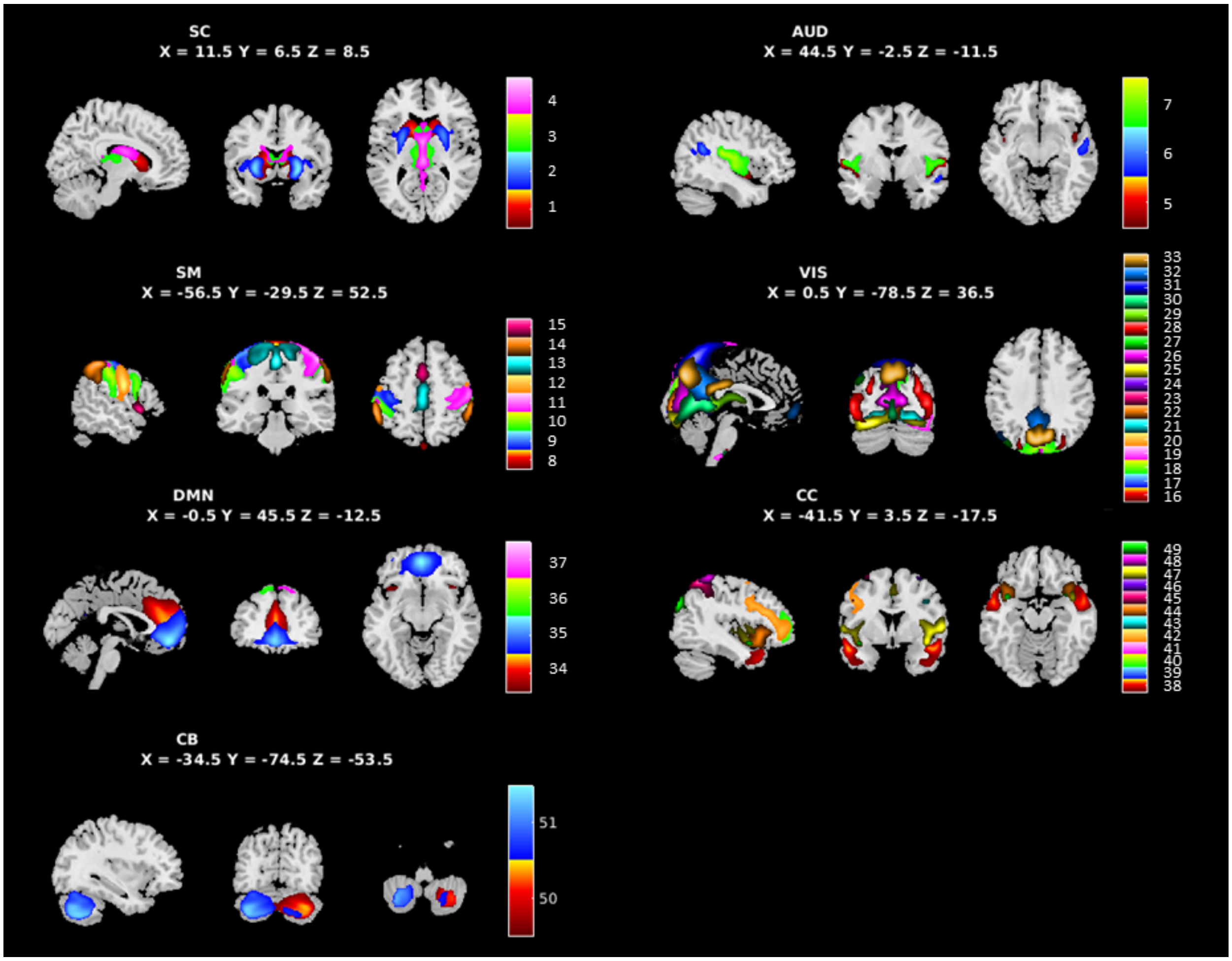

Supplementary Table 1. Component labels and coordinates of the peak activations

Components	Harvard-Oxford structural atlas	Peak (MNI)		
		x	y	z
Subcortical network (SC)				
1	Left putamen	-21	9	3
2	Right putamen	27	3	-3
3	Right Thalamus	3	-21	12
4	Left Thalamus	0	-12	12
Auditory (AUD)				
5	Heschl's Gyrus	-51	-15	9
6	Middle Temporal Gyrus, posterior division	51	-21	-9
7	Central Opercular Cortex	-39	-21	15
Sensorimotor (SM)				
8	Postcentral Gyrus	0	-33	78
9	Postcentral Gyrus	-51	-30	57
10	Central Opercular Cortex	-39	-6	15
11	Postcentral Gyrus	51	-27	57
12	Precentral Gyrus	51	-6	33
13	Precentral Gyrus	-3	-21	63
14	Supramarginal Gyrus, anterior division	-63	-39	45
15	Juxtapositional Lobule Cortex (formerly Supplementary Motor Cortex)	-3	3	63
Visual (VIS)				
16	Inferior Temporal Gyrus, temporooccipital part	-57	-45	-15
17	Occipital Pole	-21	-99	3
18	Cuneal Cortex	3	-81	33
19	Occipital Fusiform Gyrus	33	-81	-18
20	Temporal Occipital Fusiform Cortex	-30	-51	-18
21	Lingual Gyrus	15	-69	-9
22	Intracalcarine Cortex	0	-81	3
23	Cingulate Gyrus, posterior division	-9	-48	0
24	Cingulate Gyrus, posterior division	9	-45	0
25	Occipital Fusiform Gyrus	-18	-87	-18
26	Intracalcarine Cortex	3	-69	9
27	Precuneous Cortex	-15	-63	21
28	Lateral Occipital Cortex, superior division	-27	-81	24
29	Cingulate Gyrus, posterior division	-3	-45	3
30	Lingual Gyrus	-3	-63	6
31	Precuneous Cortex	0	-63	63
32	Precuneous Cortex	-3	-57	33
33	Cingulate Gyrus, posterior division	-3	-45	21
Default-mode network (DMN)				

34	Cingulate Gyrus, anterior division	-3	39	9
35	Frontal Medial Cortex	3	51	-9
36	Lateral Occipital Cortex, superior division	-51	-63	33
37	Lateral Occipital Cortex, superior division	51	-63	39
Cognitive control (CC)				
38	Middle Temporal Gyrus, anterior division	54	3	-21
39	Frontal Pole	-3	57	21
40	Frontal Pole	-30	57	3
41	Frontal Pole	-3	69	15
42	Middle Frontal Gyrus	-51	21	27
43	Insular Cortex	39	21	-3
44	Insular Cortex	-42	15	-3
45	Lateral Occipital Cortex, superior division	-27	-75	39
46	Supramarginal Gyrus, posterior division	45	-39	48
47	Central Opercular Cortex	45	3	3
48	Postcentral Gyrus	3	-51	75
49	Lateral Occipital Cortex, superior division	-54	-75	27
Cerebellar (CB)				
50	-	21	-72	-45
51	-	-33	-69	-39

Supplementary Figure 3. Values of mean dwell time (MDT, number of time windows) in each state and number of transitions between states by sex. Each point represents an observation, and each line connects two observations of the same participant.

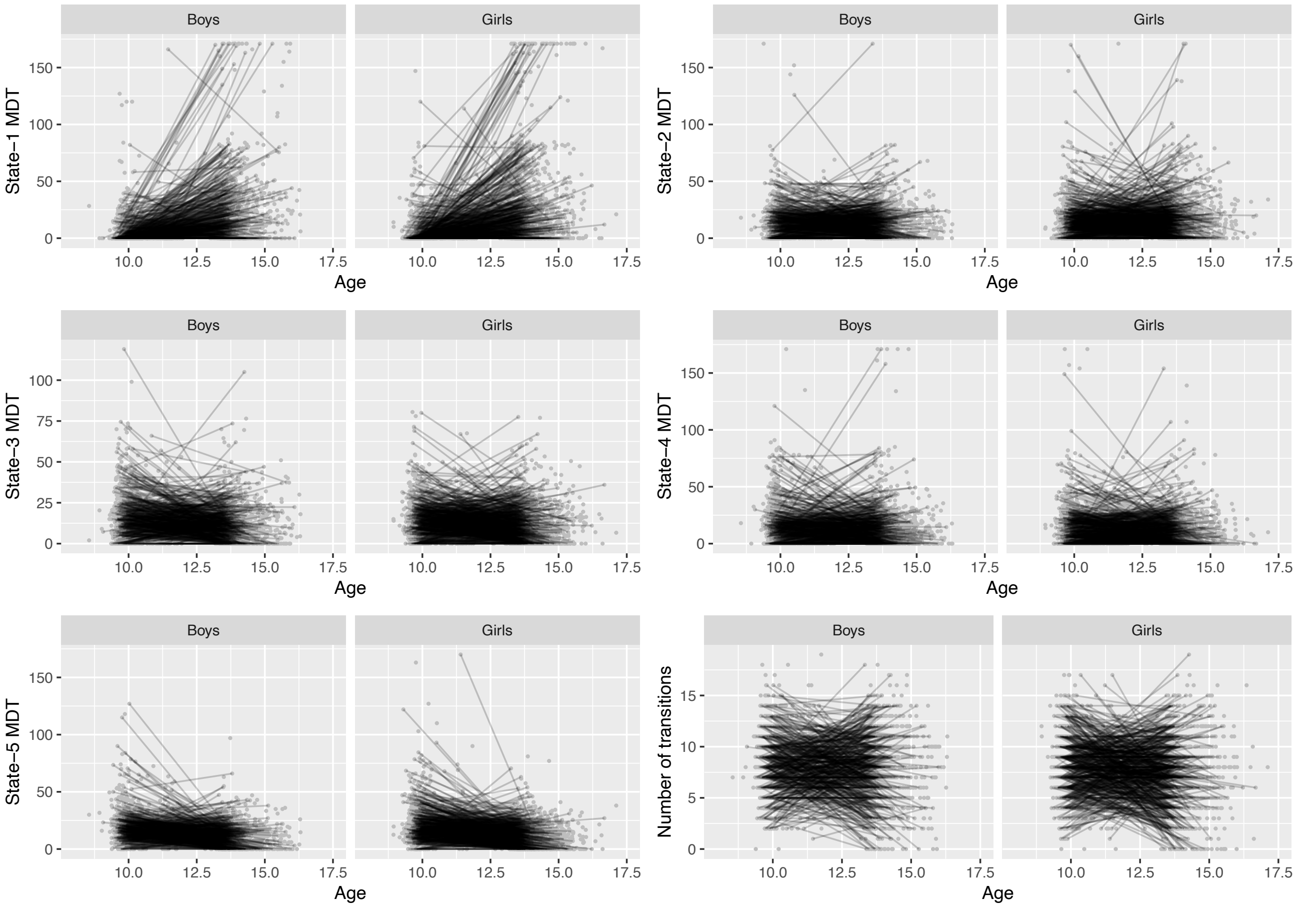

Supplementary Table 2. Age- and sex-associations with transformed mean dwell time (MDT, number of time windows) in each state and number of transitions (NT) between states adjusted for maternal education

	Age				Sex (ref. boys)				AIC	BIC
		95% CI				95% CI				
	Estimate	Lower	Upper	P value	Estimate	Lower	Upper	P value		
**State-1 MDT**	0.207	0.193	0.220	<0.001*	-0.043	-0.098	0.012	0.124	10981.04	11025.37
**State-2 MDT**	0.005	-0.010	0.020	0.523	0.146	0.085	0.207	<0.001*	11864.94	11909.27
**State-3 MDT**	-0.079	-0.094	-0.064	<0.001*	-0.073	-0.133	-0.013	0.018*	11738.10	11782.43
**State-4 MDT**	-0.037	-0.051	-0.023	<0.001*	-0.249	-0.312	-0.186	<0.001*	11735.25	11779.58
**State-5 MDT**	-0.170	-0.184	-0.156	<0.001*	0.046	-0.011	0.103	0.110	11300.56	11344.89
**NT**	-0.179	-0.225	-0.133	<0.001*	-0.228	-0.416	-0.040	0.017*	21235.53	21279.86

Linear mixed-effects models adjusted for maternal education (random effect: subject). The MDT outcomes were transformed using Box-Cox. Age was centred to the mean age of the sample at age-10 visit. *p value corrected for multiple comparisons (FDR) < 0.05. AIC = Akaike Information Criterion; BIC = Bayesian Information Criterion.
